# Concurrent radiotherapy and chemotherapy for locally advanced squamous cell carcinoma of the head and neck

**DOI:** 10.1186/1758-3284-3-48

**Published:** 2011-11-15

**Authors:** Elsayed M Ali, Ahmad G Abdelraheem

**Affiliations:** 1 Clinical Oncology department, Faculty of Medicine, Sohag University, Sohag, PO 82524, Egypt; 2 ENT department, Faculty of Medicine, South Valley University, qena, PO 83523, Egypt

**Keywords:** chemoradiation, gemcitabine, head and neck cancer, locally advanced, radiotherapy, squamous cell carcinoma

## Abstract

**Background:**

Concurrent chemoradiation is the standard treatment for patients with advanced head and neck squamous cell carcinoma (HNSCC).

The present study was carried out to assess the feasibility and efficacy of low-dose gemcitabine as a radiosensitizer when used during radical therapeutic management of patients with locally advanced HNSCC.

**Patients and methods:**

Fifty-two patients with locally advanced HNSCC (stage III, 50%; stage IVa, 50%) were enrolled during the period from July 2008 to December 2010. All received a course of radiotherapy (70 Gy over 7 weeks) concurrent with weekly infusions of gemcitabine at 50 mg/m^2^.

**Results:**

All patients were available for toxicity and response. Severe mucositis (grade 3-4) was observed in 76% of patients. Severe hematological toxicity was uncommon. Xerostomia was the most common late toxicity in 34 patients (65.4%). The rate of complete and partial response rate was 67.3% and 21.1%, respectively, with an overall response rate of 88.45%. Two years progression-free survival and disease-free survival were 46% and 38.46%, respectively.

**Conclusion:**

Using low-dose gemcitabine concurrent with radiotherapy maintains high response rate with low systemic toxicity, in spite of severe mucositis in a high percentage of patients.

## Introduction

Head and neck malignancies constitute 5% of all cancers worldwide [1]. The majority of these patients diagnosed with locally advanced disease and with lymph node involvement in up to 30-50% [2].

Locoregionally advanced head and neck carcinoma is generally treated by a combination of chemoradiotherapy, with or without surgery [3].

Because of the high incidence of advanced disease at presentation and local failure rates (50-60%), the management of head and neck carcinoma is a challenging proposition [4].

Radiation has been the standard treatment for locally advanced cancer of the head and neck. These patients, when treated with exclusive radiation, have a 5-year survival rate of less than 25%, and most treatment failures occur locally or regionally within the irradiated fields. Chemotherapy has been combined with radiation in an attempt to improve the outcome, the most promising approach being the administration of chemotherapy concurrent with radiation [4,5].

Concurrent chemotherapy and radiotherapy are widely adopted as the standard of care for locoregionally advanced squamous cell carcinoma of the head and neck after the publication of a large meta-analysis, including individual data on 10,741 patients in 63 randomized trials [6].

Many trials of concurrent chemoradiation have used cisplatin in combination with 5-fluorouracil; however, there is no evidence that this combination performs better than cisplatin alone [7].

It has been postulated that radiosensitization with gemcitabine is due to the depletion of deoxyadenosine triphosphate (dATP) through inhibition of ribonucleotide reductase by the present DNA damage caused by radiation that cannot be repaired, and this leads to an increase in cell death (Lawrence, 1996) [8].

In 1997, Eisbruch et al. reported the preliminary results of a phase I study evaluating low-dose gemcitabine concurrently with standard radiation. They found high tumor control at a dose of 300 mg/m^2 ^per week, although excessive mucosal toxicity led them to reduce the dose. Another preliminary study with 200 mg/m2 per week was performed, and encouraging response rate was observed (75% complete response), where all the patients developed grade III mucositis, and 1 patient died during treatment. Because of this, the study was terminated [9].

## Patients and methods

### Nature of the study

It is a prospective phase II non-randomized clinical trial including 52 patients with locally advanced, non-metastatic squamous cell carcinoma of the head and neck. Patients were recruited from the clinical oncology outpatient clinic at Sohag University Hospital. The study was approved by the local ethical committee of the university. All patients were given the informed consent to read, and only those who agreed to sign the consent were included.

### Eligibility criteria

• Patients with histopathologically proven squamous cell carcinoma of the head and neck.

• Stage III, IV (non-metastatic disease).

• Age more than 18 and less than 70 years old.

• WHO performance status 0, 1, or 2.

• Adequate hematological, renal, and hepatic functions.

• No prior chemotherapy or radiotherapy.

• All patients signed an informed consent.

### Pretreatment evaluation

• Clinical examination

* Including history, complete physical examination, and head and neck examination, with attention to cervical lymphadenopathy and its site, size, consistency, bilaterality, and whether fixed or mobile.

* Dental evaluation with management of dental problems and oral hygiene caring prior to starting radiotherapy.

• Laboratory

* Including complete blood count, renal and liver function tests should be done every 2 weeks and then every month during the first year.

• Endoscopic evaluation

Rigid and fibro-optic panendoscopies were performed, with mapping of the extension of the lesion for proper staging. Also, careful inspection of all mucosal lining to exclude other primary or precancerous lesions and biopsy was taken.

• Radiological

* Including CT scan for the primary site, chest x-ray, abdominal ultrasonography, and bone scan, if indicated.

### Treatment protocol

Patients received a course of radiotherapy, once daily, 2 Gray per fraction, for 5 days per week. The total dose to the macroscopic tumor and to potential sites of spread was 70 Gray, to be delivered over 7 weeks.

Concurrent chemotherapy with a course of gemcitabine was administered intravenously over 30 minutes once a week, 1-2 hours before radiotherapy, for 7 consecutive weeks, at a dose of 50 mg/m^2^.

### Evaluation during and post-treatment

All patients were clinically evaluated twice a week during treatment.

• Toxicity

Toxicity was evaluated weekly according to World Health Organization (WHO) scoring system. Any grade 4 toxicity warranted a 1-week delay in the administration of both chemotherapy and radiotherapy.

• Response criteria

Assessment of the response was performed 4-6 weeks after the end of treatment according to WHO criteria.

Tumor response was evaluated by physical examination, head and neck CT, and endoscopy with biopsy of the tumor bed.

Complete response (CR) was defined as the disappearance of all evidence of disease by physical examination, CT, and direct endoscopy.

Partial response (PR) was defined as a reduction by 50% of the product of the largest perpendicular diameters of measurable disease, with no progression at other sites of disease and no appearance of new lesions.

Tumor progression was considered if there was an increase by 25% in the product of the longest perpendicular diameters of tumor lesions or the appearance of new ones.

### Statistical analysis

Data analysis (mean, median, survival analysis, and graphs) was performed by Intercooled Stata version 9.2.

## Results

From July 2008 to December 2010, 52 patients from the clinical oncology department at Sohag University Hospital were enrolled in this trial; males were 36, and females were 16, with a median age of 54 years ranging from 25 to 70 years.

Table (1) shows baseline characteristics of study subjects. Baseline hemoglobin level ranged from 9.9 gm/dl to 15.8 gm/dl, with a median of 12 gm/dl.

**Table 1:** Baseline characteristics of study subjects

Characteristics	**No**.	%
**Sex**		
*female	16	30.77
*male	36	69.23

**Age**		
* < 50	16	30.77
* ≥ 50	36	69.23
*mean (SD)	53.73 (11.35)	
*median (range)	54 (25-70)	

**Performance status**		
*0	2	3.85
*1	24	46.15
*2	26	50

**Baseline Hemoglobin**		
*mean (SD)	12.39 (1.89)	
*median (range)	12 (9.9-15.8)	

**Site of primary tumor**		
* nasopharynx	4	7.69
* hypopharynx	8	15.38
* larynx	26	50
* maxilla	2	3.85
* paranasal sinus	2	3.85
* tongue	10	19.23

**T stage**		
*T1	4	7.69
*T2	12	23.08
*T3	20	38.46
*T4	16	30.77

**N stage**		
* N0	10	19.23
* N1	10	19.23
* N2	32	61.54

**Grade**		
*I	4	7.7
*II	34	65.4
*III	14	26.9

**Stage**		
*III	26	50
*IVa	26	50

According to ECOG classification of performance status, nearly all patients ranked in class 1 and 2, and just 2 patients ranked in class 0.

### Overall treatment time

The median overall time was 8 weeks ranging from 7 to 10 weeks. Eight patients had interruption due to severe toxicity, mainly mucositis.

### Toxicity

All 52 patients were available for toxicity; acute toxicities were common but manageable (Table 2), and mucositis and dysphagia were the most common acute toxicities. The incidence of grade 3-4 mucositis was observed in 40 patients (76.92%), grade 2 dysphagia was noticed in 22 patients (42.31%), and grade 3 dysphagia was noticed in 12 patients (23.08%).

**Table 2:** acute toxicities

Acute toxicities-grade	No. of patients	%
**Vomiting**		
0	10	19.23
1	26	50
2	14	26.92
3	2	3.85

**Dysphagia**		
0	4	7.69
1	14	26.92
2	22	42.31
3	12	23.08

**Mucositis**		
0	0	0.0
1	2	3.85
2	10	19.23
3	24	46.15
4	16	30.77

**Skin**		
0	4	7.69
1	32	61.54
2	14	26.92
3	2	3.85

Hematological toxicity was uncommon, with grade 3 neutropenia being noticed in 2 patients in week 5 during the course of treatment.

Late toxicity was observed as grade 2 xerostomia in 34 patients (65.4%). No objective evaluation of swallowing function was performed.

### Response to treatment

All 52 patients were available for response. The overall response rate was observed in 88.45% of patients. Thirty-five patients (67.30%) achieved a complete response, 11 patients (21.15%) showed partial response, and 6 patients (11.53%) showed a stationary disease. No patients had disease progression.

The median duration of response was 21 months range (4-30).

There was no correlation of response with tumor grade, nodal status, or primary site.

### Outcome

The median follow-up time was 24 months (4-30).

Five patients of 17 patients who did not achieve complete remission underwent salvage surgery, and the other 12 patients received palliative chemotherapy.

Three patients of the 35 patients, who had achieved complete remission, relapsed after 1 year of follow-up, 1 patient underwent salvage surgery, and 2 patients refused surgery and received chemotherapy.

**Figure 1:**
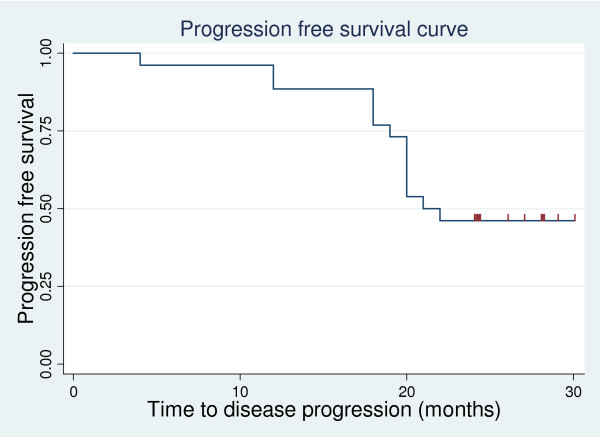
**progression- free survival curve**.

**Figure 2:**
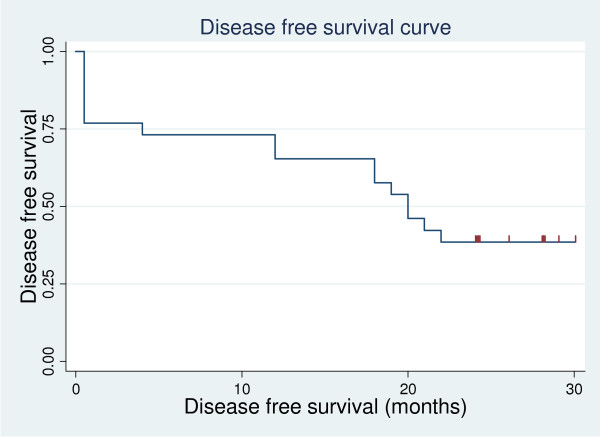
**disease -free survival curve**.

The median time of disease progression was 21.5 months (4-30), with 2 years progression-free survival (PFS) of 46% (Figure 1), and the median disease-free survival (DFS) was 20 months (0-30), with 2 years DFS of 38.46 (Figure 2).

## Discussion

Although concurrent chemoradiation has become the standard of care for advanced and/or unresectable head and neck carcinoma patients, the best drug and schedule for chemoradiotherapy remains to be undermined.

This trial was designed to test the efficacy and toxicity of a regimen of weekly gemcitabine, concurrent with radiotherapy in patients with advanced HNSCC. Thirty-six (68.5%) patients had T3 and T4 nodes, and 32 (61.5%) patients had N2 nodes. Despite these unfavorable patients' characteristics, this regimen showed an encouraging tumor response rate and acceptable survival results.

In the present study, we achieved an overall response rate of 88.45% (67.30% complete response, 21.15% partial response). This result is in agreement with the result reported by Aguilar et al., 2004, who achieved an overall response rate of 88%. It is also comparable with the result reported by Eisbrush et al., 2001, who achieved 66% complete response rate [10,9].

We observed a significant rate of grade 3-4 mucositis (76%). This was in agreement with the result of Aguilar et al., 2004, in which 74% of grade 3-4 mucositis was reported, and also with Shaharyar et al., 2006, who reported 77% of grade 3-4 mucositis in their study [10,11].

In our study, the hematological toxicity was mild, and grade 3 neutropenia was observed in 2 patients; this result is comparable to what was reported by Eisbruch et al., 2001, and Aguilar et al., 2004 [9,10].

In our study, the median time to disease progression was 21.5 months, with 2 years PFS of 46%, and the median disease-free survival was 20 months, with 2 years DFS of 38.46%. Our result is lower than that of Chauhan et al., 2008, and this may be because the median follow-up time is longer in our study compared with their study, which was 11 months [12].

## Conclusion

The concurrent use of radiotherapy and low-dose gemcitabine demonstrated an encouraging response rate as compared to other chemoradiation trials.

Gemcitabine at relatively low doses is a potent radiosensitizer in HNSCC patients, but it produces a high incidence of mucositis. Further studies are needed to optimize the administration of gemcitabine with radiation.

## Abbreviations

HNSCC: head and neck squamous cell carcinoma; PFS: progression-free survival; DFS: disease-free survival.

## Competing interests

The authors declare that they have no competing interests.

## Authors' contributions

EM, have been carried out and drafted the manuscript. Also he has been revised it critically for important intellectual content. AG, participated in its design.

All authors read and approved the final manuscript.
